# Southern Medical College Advertisement

**Published:** 1889-07

**Authors:** 


Read the Southern Medical College advertisement; also the advertisement of the Dental Department of this school.

